# Hookah-Related Twitter Chatter: A Content Analysis

**DOI:** 10.5888/pcd12.150140

**Published:** 2015-07-30

**Authors:** Melissa J. Krauss, Shaina J. Sowles, Megan Moreno, Kidist Zewdie, Richard A. Grucza, Laura J. Bierut, Patricia A. Cavazos-Rehg

**Affiliations:** Author Affiliations: Shaina J. Sowles, Kidist Zewdie, Richard A. Grucza, Laura J. Bierut, Patricia A. Cavazos-Rehg, Department of Psychiatry, Washington University School of Medicine, St. Louis, Missouri; Megan Moreno, Seattle Children’s Research Institute, Center for Child Health Behavior and Development, Seattle, Washington.

## Abstract

**Introduction:**

Hookah smoking is becoming increasingly popular among young adults and is often perceived as less harmful than cigarette use. Prior studies show that it is common for youth and young adults to network about substance use behaviors on social media. Social media messages about hookah could influence its use among young people. We explored normalization or discouragement of hookah smoking, and other common messages about hookah on Twitter.

**Methods:**

From the full stream of tweets posted on Twitter from April 12, 2014, to May 10, 2014 (approximately 14.5 billion tweets), all tweets containing the terms hookah, hooka, shisha, or sheesha were collected (n = 358,523). The hookah tweets from Twitter users (tweeters) with high influence and followers were identified (n = 39,824) and a random sample of 5,000 tweets was taken (13% of tweets with high influence and followers). The sample of tweets was qualitatively coded for normalization (ie, makes hookah smoking seem common and normal or portrays positive experiences with smoking hookah) or discouragement of hookah smoking, and other common themes using crowdsourcing.

**Results:**

Approximately 87% of the sample of tweets normalized hookah use, and 7% were against hookah or discouraged its use. Nearly half (46%) of tweets that normalized hookah indicated that the tweeter was smoking hookah or wanted to smoke hookah, and 19% were advertisements/promotions for hookah bars or products.

**Conclusion:**

Educational campaigns about health harms from hookah use and policy changes regarding smoke-free air laws and tobacco advertising on the Internet may be useful to help offset the influence of pro-hookah messages seen on social media.

## Introduction

Hookah smoking is popular among college-aged students and recently has nearly doubled among adolescents in the United States (1,2). This increase comes despite the public health achievement of decreased cigarette use in this population over the past several decades (3). Despite research demonstrating hookah use exposes smokers to toxins similar to those in cigarette smoke and increases exposure to carbon monoxide (4,5), hookah use is widely perceived as less harmful (6,7).

Hookah smoking does not carry the same stigma as cigarettes; to the contrary, hookah use promotes social interaction and is perceived as trendy and fun (8). Social media posts are known to portray substance use, like marijuana and alcohol, as normative (9–11) or allow underage users to easily see endorsements (12). However, few studies have investigated discussion of hookah use on social media. One study examined the Facebook accounts of a sample of 307 college students and found that references to hookah smoking were found in 5% of the accounts (13). A Twitter-based study of tobacco-related discussion found that personal experiences and opinion were the most common subjects, and approval was commonly expressed when referencing electronic cigarettes and hookah (14). Pro-substance use messages on social media can further normalize use among the audience (10,11). Repeated exposure to this approval will strongly influence the attitudes and behaviors of those exposed to them.

In this exploratory study, we examined Twitter chatter about hookah. Although Twitter is one of the most popular social networking sites among young adults (15), no studies have focused solely on discussion of hookah use on Twitter. We examine hookah-related Twitter chatter for normalization vs discouragement, themes, and commercial promotions behind the chatter. This knowledge can help inform public health organizations about how to combat the growing trend of hookah use among young people.

## Methods

The data used in this study are publicly available via Twitter and do not contain identifiers other than the Twitter username. Thus, our study does not involve human subjects and is not subject to institutional review board jurisdiction. The flow of the methodology used for our study is summarized in the Figure.

**Figure:**
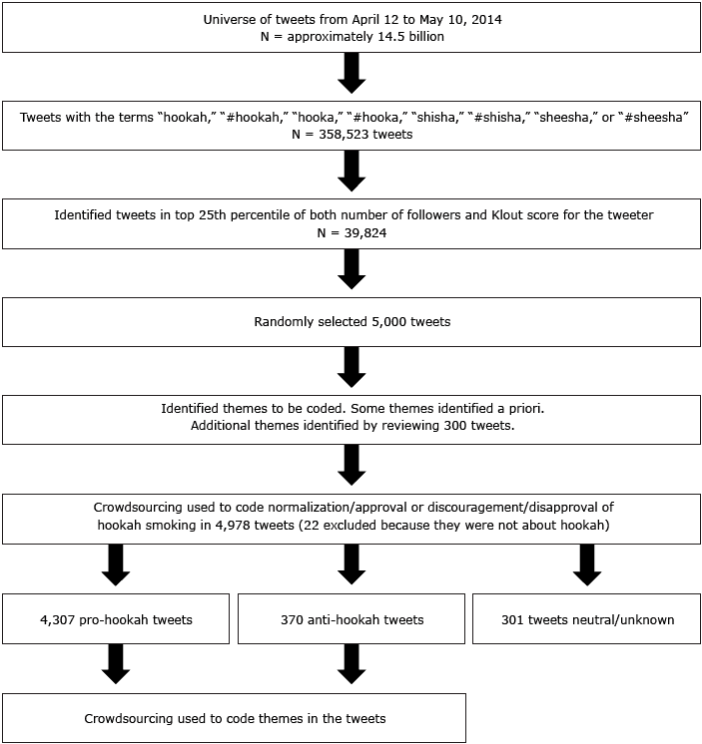
Methodology of a content analysis of hookah-related Twitter chatter.

We collected all tweets in English that contained the terms hookah or #hookah, shisha or #shisha, as well as common alternative spellings of these terms (ie, hooka, #hooka, sheesha, #sheesha) from April 12 to May 10, 2014, using Simply Measured (http://simplymeasured.com/), a social media analytics company with access to 100% of Twitter’s Tweets (the “Firehose”). The terms are the most commonly used for hookah according to Google Trends (http://www.google.com/trends/), a site that depicts the popularity of search terms in Google, and Topsy (http://topsy.com/), a site that estimates the total volume of tweets with specific terms over the past month. Searching for these terms would also include tweets mentioning electronic hookah devices (eg, hookah pens, e-hookah). Although these electronic nicotine delivery systems have different health risks than traditional hookah, they are growing in popularity and we did not want to exclude them from this study.

Simply Measured also provides meta-data for the tweets, including number of followers for each of the Twitter handles associated with the tweets and the Klout score associated with the handle of the Twitter user who tweeted the message. Klout score ranges from 1 to 100 and indicates how influential a user is on social media; the higher the number, the greater the influence (16).

### Qualitative analysis of the tweets

To focus on tweets with the most influence, we identified tweets in the top 25th percentile of both number of followers and Klout score. Although these measures are correlated (Spearman’s *r* = 0.57), number of followers indicates popularity and Klout score measures the ability to drive action and takes many signals into consideration (eg, ratio of user-generated reactions compared to the amount of content the user shares) (16). We examined tweets in the top 25th percentile, rather than a smaller percentile (eg, 10th), to include influential handles beyond popular celebrities or news-focused handles who tend to have some of the highest follower counts. We also excluded tweets that were direct @replies to another Twitter user because these replies can be hard to discern without additional conversational information. From tweets with high number of followers and high Klout score, we randomly selected 5,000 tweets for qualitative analysis. A total of 5,000 tweets would allow estimation of the percentage of pro- and anti-tweets with 95% confidence level with a margin of error of plus or minus 1%, assuming levels of pro- and anti-sentiment similar to those seen in marijuana-related tweets (10).

*A priori*, the research team decided that each tweet would be coded for normalization or discouragement of hookah using a Likert scale: 1, strongly against (anti) hookah; 2, slightly against (anti) hookah; 3, neutral/unknown; 4, slightly normalizes/promotes hookah; 5, strongly normalizes/promotes hookah. Anti-hookah tweets discourage the use of hookah or reflect unpleasant experiences or feelings about its use. Pro-hookah tweets make hookah smoking seem common and normal, portray positive experiences with smoking hookah, or encourage its use. Tweets with neutral or unknown sentiment were excluded from additional qualitative analysis. Although the Likert scale was provided during coding to allow the distinction between tweets with strong versus slight normalization or discouragement, during analysis the responses were aggregated to only distinguish the anti-, neutral, and pro-hookah tweets (see description of aggregation methods below).

In addition to coding tweets as pro- or anti-hookah smoking, we analyzed thematic content to identify themes, or expressions of concepts, that recurred in the pro- and anti-hookah tweets (17). Some themes were determined *a priori*, based on our prior inductive examination of marijuana-related tweets (10). Additional themes were identified from the hookah tweets themselves, using the constant comparison method (18). Two members of the research team scanned approximately 300 tweets to identify additional themes. Both commercial and noncommercial tweets were included to examine how much of the influential tweets advertised a bar/product and how much appeared to be noncommercial or from regular people who were not advertising bars/products. The final list of pro-hookah themes included 1) the tweeter is smoking hookah or wants/plans to smoke hookah; 2) the tweeter recently used hookah; 3) the tweet is an advertisement for hookah products or an advertisement/promotion of hookah at bars/lounges/events; 4) the tweet mentions a song/music about hookah; 5) the tweet mentions other substances (ie, alcohol, marijuana, other drugs), 6) the tweet mentions sex or romance; 7) the tweet mentions electronic hookah or hookah pens. The final list of anti-hookah themes were 1) the health harms and other negative effects of hookah; 2) hookah smoking is unattractive, uncool, or disgusting; 3) quitting smoking hookah; 4) the tweeter doesn’t smoke hookah and/or doesn’t want to try smoking hookah; and 5) the tweeter prefers to use a different substance, such as marijuana, as opposed to hookah smoking. A tweet could convey more than one theme.

After identifying relevant themes, we used crowdsourcing, which involves using a large number of online workers to complete micro-tasks, to code the sample of 5,000 tweets. We used CrowdFlower (http://www.crowdflower.com), an online company whose platform manages an on-demand, online workforce. We uploaded the sample of tweets onto CrowdFlower’s online platform and included detailed instructions to educate CrowdFlower’s contributors (online workers) about hookah and what types of tweets fall into the specified themes. The research team coded 200 tweets to use as test items for the CrowdFlower contributors. Before beginning to code, a contributor had to score 80% or higher on 10 test items. Then, as the contributor coded tweets, test items were interspersed throughout the job to assure that the contributor continued to code at a high quality. Contributors who failed to maintain a high “trust” score (≥80% on test items) were dropped from the job, all of their prior responses were disregarded, and a different contributor was assigned to code those tweets.

At least 3 CrowdFlower contributors coded each tweet. For normalization/approval versus discouragement/disapproval, which was assessed on a Likert scale, the average score across contributors was used. Then the average scores were collapsed into anti-hookah (scores 1.0–2.4), neutral/unknown (scores 2.5–3.4), and pro-hookah (scores 3.5–5.0). For the presence of specific themes (coded as yes/no), the response with the highest confidence score was chosen. Confidence score describes the level of agreement between multiple contributors, is weighted by the contributors’ trust scores, and indicates “confidence” in the validity of the result (http://success.crowdflower.com/customer/portal/articles/1295977-how-to-calculate-a-confidence-score).

Members of the research team coded a random sample of 200 tweets that were not used as test items and compared responses to those from CrowdFlower contributors. Interobserver agreement was good for pro- versus anti-hookah (intraclass correlation 0.76), pro-hookah themes (median κ, 0.85; range, 0.65–1.00), and anti-hookah themes (median κ, 0.66; range, 0.41–1.00). The anti-hookah theme “not smoking hookah/not wanting to try hookah” had κ of 0.41, but percent agreement for this theme was 80%; thus, we decided to keep this theme in our report. After CrowdFlower coding was complete, the research term coded the source of tweets that advertised hookah bars/products as from a) commercial hookah establishments (ie, bars/clubs with hookah, hookah retailors), b) disc jockeys (DJs), club/event/local entertainment promoters, or c) other source/unknown.

## Results

From April 12 to May 10, 2014, we garnered 358,523 total tweets with our hookah key words of interest. The median Klout score for people who tweeted was 40.0 (interquartile range [IQR] 33.3–43.9) and the median number of followers was 404 (IQR 186–874).

From the 39,824 tweets from people in the top 25th percentile of both Klout score (≥43.9) and number of followers (≥874) (and were not direct @replies), we randomly selected 5,000 tweets for qualitative analysis. Of the 5,000 tweets selected for qualitative analysis, only 22 (0.4%) could not easily be identified as about hookah smoking (eg, used “hooka” as slang for “hooker” or the tweet was too difficult to understand) and were excluded from further analysis. Thus, 4,978 tweets were qualitatively analyzed.

Of the 4,978 tweets, a majority (87% or 4,307) normalized hookah or promoted its use, and only 7% (370) were against hookah or discouraged its use. Approximately 6% (301) were neutral or could not be discerned (Table 1). When considering the followers of only unique tweeters within each classification, the potential reach, or sum of the followers of the tweets, was 14 times higher for tweets that normalized hookah (4,307 tweets; 3,147 unique tweeters; 21,671,270 followers) than for those that were against hookah (370 tweets; 360 unique tweeters; 1,536,432 followers).

**Table 1:** Sentiment and Reach of a Sample of Hookah-Related Tweets With High Klout Scores and Number of Followers (Tweets, N = 4,978)^a^

Sentiment	Tweets, N (%)	No. of Unique Tweeters	Potential Reach^b^
Normalizes/Promotes hookah	4,307 (87%)	3,147	21,671,270
Against/Discourages hookah	370 (7%)	360	1,536,432
Neutral/Unknown	301 (6%)	297	1,671,190

a Randomly sampled from 39,824 hookah-related tweets with ≥874 followers and Klout score ≥43.9.

b Sum of followers of unique tweeters.

We present the number and percentage of themes among the 4,307 tweets that normalized or promoted hookah use (Table 2). Nearly half (n = 1,975, 46%) indicated that the tweeter was smoking hookah or wanted to smoke hookah. Over a quarter (n = 1,160, 27%) were tweets that advertised hookah at bars/lounges/events or advertised specific hookah products. Among these, most (85%, or n = 982 of 1,160) promoted hookah at bars/clubs/events; 60% (n = 592/982) of these were from DJs or club/event/entertainment promoters, 8% (n = 78/982) were directly from bars/lounges, and 32% (n = 312/982) were from other Twitter users or the type of tweeter could not be determined. Approximately 12% (n = 134/1,160) of the advertising tweets promoted the sale of specific hookah products. Approximately 19% (n = 837) of the tweets that normalized hookah were related to songs about hookah. Other substances (ie, alcohol, marijuana, other drugs) were mentioned in 15% (n = 625) of the tweets. Approximately 9% of the tweets mentioned hookah pens or e-hookahs. Additional themes are shown (Table 2).

**Table 2:** Themes Among Tweets That Normalize or Promote Hookah (N = 4,307)

Themes	N (%)	Sample Tweets
Tweeter is smoking hookah or wants to smoke hookah	1,975 (46%)	I want hookah. Smokin da hookah with good homies Hookah sounds like a great idea tonight.
Marketing/promotion of hookah bars/lounges, availability of hookah at bar/club, or advertising hookah products	1,160 (27%)	Weekend is not over yet! Make sure you check out #SelectSunday tonight! Great Drinks, Wings, and Hookah! Bang! Only 18+ Event Saturday Night So Come Turn Up! Get Drunk And Smoke Hookah And Party Party 4544 South Blvd ATOMIC EVOD 650 MAH MT3 ATOMIZER, COLOR STEEL, BEST PRODUCT E HOOKAH, TOP SELLER: Price 12.89 USD
Tweet is about a song/music about hookah	837 (19%)	Baby pass me the Hookah Smokin on a tooka like it’s hookah now every time i hear that song “hookah” i gotta do the dance
Mentions alcohol, pills, marijuana, other drugs	625 (15%)	I want weed and hookah. The cool part about working at a hookah lounge is getting to mix the ganja and shisha together while on duty Hookah and vodka happiness
Mentions new products like hookah pens, e-hookahs, etc.	385 (9%)	Really need a hookah pen Getting my E-Hookah soon my friends friend is buying me a hookah pen im happy
Mentions sex, romance, or attraction	106 (2%)	I forgot to say I like going on hookah dates too Let’s smoke hookah and make out or some shit like that Hookah makes hoes horny lol
Tweeter used hookah in the recent past (eg, last night, last weekend, in the past week)	78 (2%)	Went and did hookah last night with my roommate and this girl Alison. All she talked about was her bf. like, you’re too cool for that... at least we were smoking shisha pens and literally still can taste it now but I was drunk and did hookah like last week so I can’t say much

Among the 370 tweets that were against hookah smoking, two-thirds (66%) indicated that the tweeter thought that hookah use was unattractive, uncool, or disgusting. Approximately 20% (n = 74) emphasized that hookah use is harmful to your health or can have negative effects. Ten percent (n = 36) specified that the tweeter preferred another substance, like marijuana, over hookah. Additional anti-hookah themes are presented (Table 3).

**Table 3:** Anti-Hookah Themes in Tweets (N = 370)

Themes	N (%)	Sample Tweets
Hookah is gross, unattractive, not cool, stupid	246 (66%)	hookah is so childish Smoking hookah looks so dumb to me. Whats the fuckin point. Shisha has to be the dumbest alternative to people who claim to be “non-smokers”
Hookah use is harmful to your health, or can have negative effects (headache, sick, nauseous)	74 (20%)	Wow! Hookah contains 200 times more smoke than cigarettes! Hookah makes me light headed af Why do people smoke hookah hahahaha #WasteOfALung
Tweeter prefers other substances over hookah (eg, marijuana)	36 (10%)	Fuck hookah... just smoke a blunt lol Friends dont let friends get fake high off hookah... Pass them real drugs Hate hookah where the weed at?
Tweeter doesn’t use hookah or doesn’t want to try it	28 (8%)	i don’t smoke, weed, drugs, shisha, or drink (never even tried any) Most people are like “as soon as I'm 18 I’m going to a strip club, hookah bar, etc.” Then there is me who is gonna go skydiving and such Vape or hookah is so pointless to me... but hey, that’s just me
Quitting hookah use	10 (3%)	I’ve been hookah free for 2 months now feels so good! I don’t want to see liquor or hooka for a while I don’t wanna smoke hookah or weed for a while man

## Discussion

Our study, the first to focus solely on tweets about hookah use, demonstrates that most hookah-related tweets from Twitter users with high influence and followers are pro-hookah. On the basis of total volume of hookah-related tweets collected over nearly a month in our study (n = 358,523/29 days), we estimate that more than 12,000 hookah-related tweets are sent each day (about 500 million tweets are posted daily on Twitter). This is considerably fewer than tweets about marijuana (over 250,000/day) or alcohol (at least 400,000/day) (10,11), but substantially more than tweets about e-cigarettes found in 2012 (1200/day) (19). In addition, hookah use is a growing trend that has doubled among middle and high school students in just 1 year (2,20). Consequently, the social networking about hookah is likely to increase as well.

Nearly 90% of the tweets in our sample normalized or promoted hookah, and these had much higher potential reach (or sum of all followers) than anti-hookah tweets. Similarly, a prior study of tobacco-related tweets that used machine-learning algorithms to classify tweets (ie, using natural language processing techniques via computers to automatically classify tweets) found a high prevalence of positive sentiment toward tobacco products, and hookah was one of the prominent themes (14). It is evident that young people are willing to broadcast their experiences with hookah openly on social media. Peer smoking behaviors are important influences on youth tobacco use behaviors (21). In this age of social media, peer influences extend beyond those in our proximate communities to those in our virtual social networks. The proliferation of pro-hookah messages on social media could impact social norms, further influencing more young people to try hookah.

Nearly a quarter of the pro-hookah tweets were commercial promotions of hookah at bars/clubs/events, and such venues are likely to facilitate the social aspect of hookah smoking that appeals to young people (22). Despite the widespread adoption of smoke-free air laws throughout the United States, hookah bars are often exempt from these laws (23). In fact, a study of smoking bans in the 100 largest US cities found that 73 banned cigarette smoking in bars but 69 of these cities had exemptions that allowed hookah smoking (24). Integrating hookah smoking within smoke-free air laws could help to reduce exposure to secondhand hookah smoke and establish norms against hookah smoking (25).

Social media is an important part of a marketing strategy for businesses, allowing companies to network directly with potential customers and for others to network with each other about companies or products (26). As seen in our study, promotional messages for hookah can come from hookah commercial entities and entertainment promoters. Although hookah bar advertisements usually target customers who reside near these venues, hookah-related establishments are emerging in communities throughout the United States, especially around college campuses (1). Hookah-related industries are likely to use social media similarly to advertise their services to a mass audience of potential consumers. Given that other types of tobacco have also been promoted through online media, exploring the possibilities of online tobacco marketing restrictions may be necessary (27).

Several tweets mentioned e-hookah or hookah pens. Similar to e-cigarettes, these small portable electronic devices vaporize a liquid that is inhaled. Twitter is already an important medium to market e-cigarettes (19). Although these products may be less harmful than smoking regular cigarettes or hookah, limited data suggest that they are not harmless (28). Such products are enticing to youth and could lead young people to use conventional tobacco products (29). As social media networking about these products increases among youth and young adults, awareness and use of e-hookah will probably increase as well.

About 15% of the pro-hookah tweets mentioned using other substances in addition to hookah smoking. In fact, hookah smokers also tend to use other tobacco products, drink alcohol, or smoke marijuana (7). Given that combined use of tobacco and alcohol greatly increases the risk of mouth and throat cancers, and that use of tobacco and marijuana increases the risks of respiratory health effects, it is worrisome that messages normalizing polysubstance use are being communicated to wide audiences via Twitter (30,31).

Very few tweets (only 7%) expressed opinions against hookah, and these infrequent messages tended to be about the health harms or the belief that hookah smoking is uncool or unattractive. Hookah smokers may absorb greater concentrations of toxins than cigarette smokers because of the length of the smoking session and the depth of inhalation, and hookah smoking is associated with respiratory problems, lung cancer, and other negative health effects (4). However, many young people view hookah smoking as less harmful than smoking cigarettes (7). Both online and offline educational strategies are needed to counter the widespread pro-hookah messages that young people view and to effectively communicate the health harms associated with hookah smoking.

Our study is not without limitations. We captured only tweets with the terms hookah and shisha (and common alternative spellings) from approximately 1 month. Although these are probably the most common terms used, an examination of a more exhaustive list of hookah-related terms (eg, narghile, waterpipe) over a longer period of time could provide a more detailed analysis of attitudes and themes about hookah over time. Our results might not be generalizable to users of other social media sites, as we examined only Twitter. Our qualitative analysis was limited to a subgroup of tweets with high influence and high number of followers; sentiment and common themes could differ for tweets with lower influence and followers. Finally, people with negative attitudes about hookah smoking may also be less likely to use Twitter.

Our novel findings suggest that tweets normalizing or promoting hookah smoking are common on Twitter and reach a large audience. Many of the pro-hookah tweets are noncommercial (ie, from people unassociated with the business) and express currently smoking/wanting to smoke hookah or mention electronic hookah products and polysubstance use. Furthermore, commercial advertisements for hookah bars and hookah products were also common. Such messages may influence the social norms of the followers of these tweets and further increase hookah smoking among young people. To help offset these pro-hookah influences, public health campaigns are needed to effectively inform youth and young adults about the serious health harms of hookah use. Moreover, amending smoke-free air laws to eliminate exemptions for hookah bars and exploring the possibilities of restricting tobacco advertising on the Internet are prudent.
